# Supplementary material for: Targeting Tat–TAR RNA Interaction for HIV-1 Inhibition
Source: Viruses. 2021 Oct 6;13(10):2004. doi: 10.3390/v13102004 (PMC8536978; doi:10.3390/v13102004)
Supplement: Supplementary file 1 [file viruses-13-02004-s001.zip › viruses-1356678-supplementary.pdf]

Supplemental Figures

# Targeting Tat-TAR RNA Interaction for HIV-1 Inhibition

Awadh Alanazi, Andrey Ivanov, Namita Kumari, Xionghao Lin, Songping Wang, Dmytro Kovalskyy and Sergei Nekhai

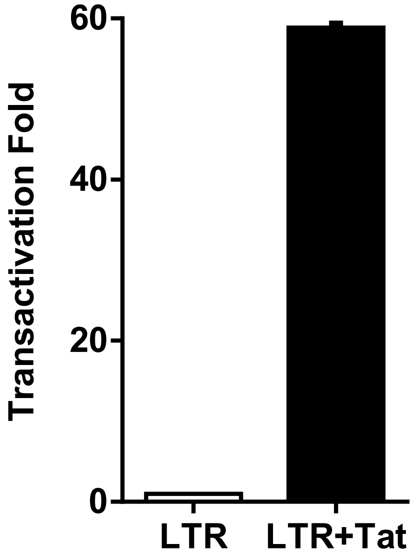


**Supplemental Figure S1. Activation of HIV-1 transcription by Tat.** 293T cells were transfected with vectors expressing HIV-1 LTR-*Luciferase* (lane 1) or HIV-1 LTR-*Luciferase* and HIV-1 Tat expression vectors (lane 2). After 24 hours of culturing, the cells were lyzed and luciferase activity was determined using LuciLite assay.

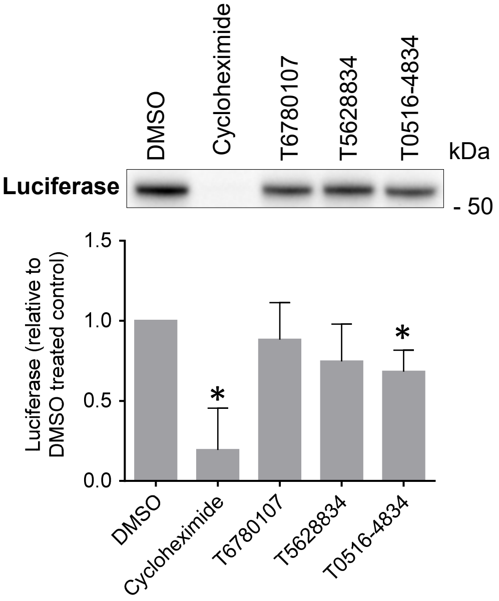


**Supplemental Figure S2. Effect of Tat-TAR RNA targeting compounds on luciferase translation.** Luciferase mRNA was translated in vitro rabbit reticulocyte lysate using non-radioactive reticulocyte translation system with ε-labeled biotinylated lysine-charged $tRNA^{Lys}$. Reactions were carried as describe in Materials and Methods and resolved on SDS-PAGE. Asterisks indicate $p<0.05$.

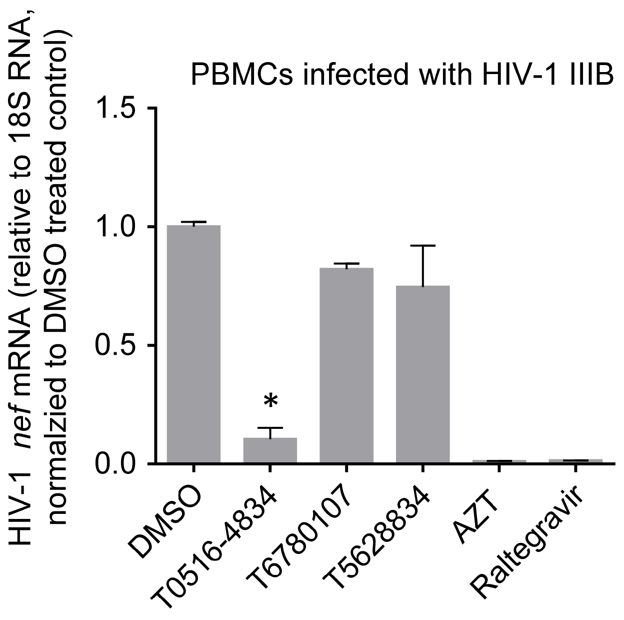


**Supplemental Figure S3. Inhibition of HIV-1 replication in PBMCs infected with HIV-1 IIIB.** PBMCs were activated as described in Materials and Methods and infected with HIV-1 IIIB for 48 hrs followed by RNA isolation and real-time PCR analysis for HIV-1 *nef* mRNA. Asterisk indicate $p<0.05$ in comparison to the DMSO controls.

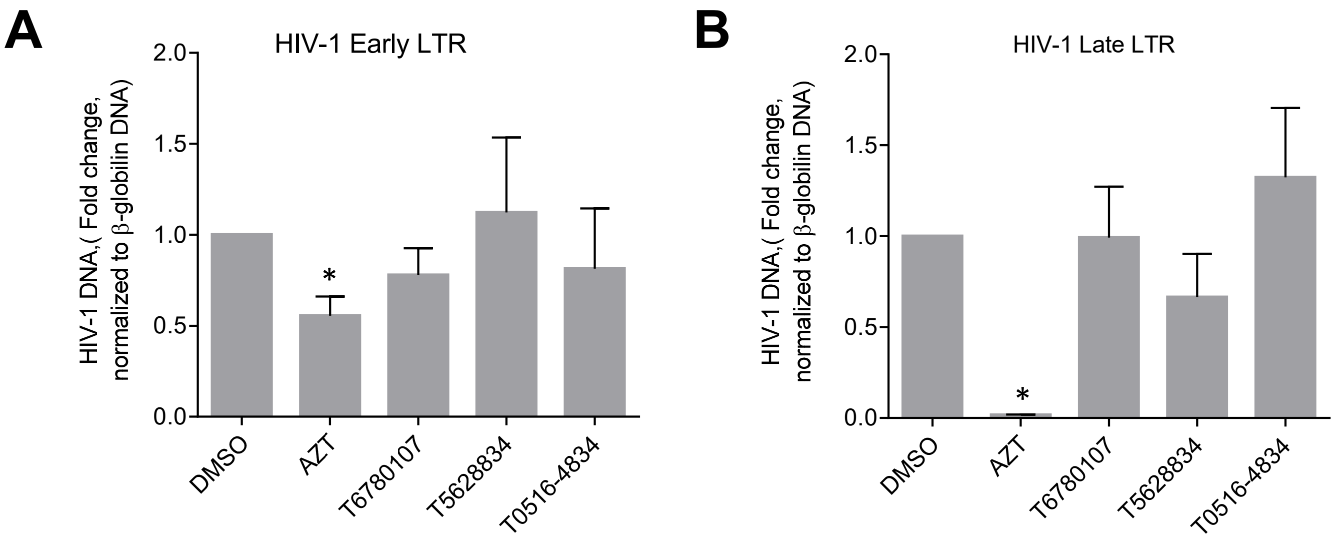


**Supplemental Figure S4. No effect of Tat-TAR RNA targeting compounds on HIV-1 reverse transcription.** PBMCs were activated with PHA and IL-2 and infected with HIV-1-LUC-G. At 6 hrs p.i. DNA was extracted and analyzed by real-time PCR on Roche 4800 using primers for early LTR (panel A) and late LTR (panel) and β-globin gene as a reference. Azidothymidine (AZT) was used as control. The means ± SD are shown (n=3 for each sample). Asterisk indicate $p<0.05$.

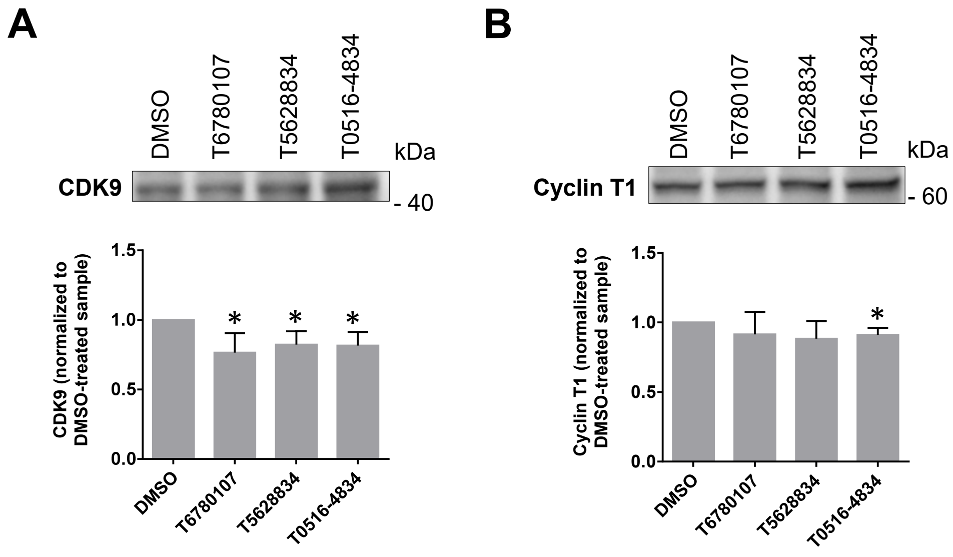


**Supplemental Figure S5. No effect of TAR RNA targeting compounds on CDK9 and cyclin T1 protein levels**. 293T cells were treated with 20 μM compounds, lysed and proteins were resolved on 10% SDS-PAGE. A, CDK9 protein expression determined using anti CDK9 antibodies. B, cyclin T1 expression determined by anti-cyclin T1 antibodies. Quantification was performed using Prism 6 from three independent experiments. Asterisks indicate $p<0.05$ in comparison to the DMSO controls.

**Supplemental Table S1.** Data plotted on Fig.1 from the high throughput screening of TAR RNA-targeting small molecules for one round HIV-1 infection. DMSO represents 100% and percentage of inhibition was calculated for each compound.

| Compound | Luciferase (%, relative to DMSO control) | SD |
|---|---|---|
| **DMSO** | 100.000000 | 7.755011 |
| **T0505-5074** | 83.440540 | 7.268419 |
| **T0506-3768** | 83.570630 | 6.594526 |
| **T0512-9025** | 128.440800 | 23.355570 |
| **T0513-0728** | 138.721400 | 7.808097 |
| **T0516-4834** | 37.736560 | 1.991454 |
| **T0516-7553** | 137.585100 | 9.868014 |
| **T0518-6335** | 134.383000 | 4.015627 |
| **T0518-7949** | 131.267400 | 14.930890 |
| **T0519-2369** | 56.074370 | 7.556275 |
| **T0520-2423** | 48.833160 | 6.437354 |
| **T5267033** | 73.165430 | 4.672985 |
| **T5276690** | 174.325400 | 7.098480 |
| **T5284151** | 154.157200 | 5.171193 |
| **T5311624** | 137.579700 | 6.066707 |
| **T5324124** | 162.556800 | 5.436389 |
| **T5325151** | 163.256400 | 13.804960 |
| **T5328103** | 133.752800 | 13.968430 |
| **T5333529** | 122.959100 | 9.202515 |
| **T5334449** | 167.494300 | 3.017701 |
| **T5335152** | 18.259700 | 1.710728 |
| **T5338009** | 78.355870 | 5.993520 |
| **T5338449** | 162.628200 | 6.500543 |
| **T5349850** | 140.771700 | 4.836438 |
| **T5351437** | 153.402100 | 6.488094 |
| **T5370484** | 140.326300 | 12.618240 |
| **T5374886** | 121.703600 | 5.993004 |
| **T5377732** | 83.101330 | 3.448379 |
| **T5387947** | 53.505550 | 5.494543 |
| **T5412447** | 189.647400 | 10.474050 |
| **T5414017** | 219.124400 | 11.131290 |
| **T5435009** | 165.879300 | 4.646722 |
| **T5435809** | 96.220200 | 6.874708 |
| **T5452365** | 119.463100 | 6.354763 |
| **T5461591** | 107.366800 | 4.328526 |
| **T5463484** | 89.543080 | 4.301324 |
| **T5477469** | 102.062800 | 12.980950 |
| **T5478937** | 101.797100 | 3.227994 |
| **T5490993** | 96.382140 | 4.706879 |
| **T5500667** | 131.740400 | 6.452964 |
| **T5503929** | 153.784500 | 8.088698 |
| **T5522686** | 93.525230 | 4.736200 |
| **T5540730** | 119.695500 | 11.462510 |
| **T5543348** | 96.630670 | 13.387070 |
| **T5546471** | 187.615800 | 12.917400 |
| **T5562017** | 41.862260 | 2.851782 |
| **T5562906** | 108.776900 | 2.070146 |
| **T5565384** | 112.892900 | 6.129734 |

| | | |
|---|---|---|
| **T5568878** | 115.583600 | 0.736923 |
| **T5571459** | 71.713370 | 5.492987 |
| **T5574832** | 85.155750 | 2.961138 |
| **T5583064** | 29.059050 | 1.904799 |
| **T5588257** | 97.217670 | 6.983262 |
| **T5589604** | 116.198900 | 16.495400 |
| **T5602702** | 114.090800 | 11.607730 |
| **T5628834** | 28.711770 | 0.995325 |
| **T5630140** | 92.124890 | 9.143012 |
| **T5631558** | 118.464300 | 9.133316 |
| **T5635515** | 99.655270 | 4.679419 |
| **T5648026** | 106.288900 | 7.976184 |
| **T5653944** | 64.939710 | 7.337393 |
| **T5656438** | 37.823840 | 0.138798 |
| **T5658369** | 104.304600 | 20.987490 |
| **T5668298** | 110.355900 | 8.054684 |
| **T5683862** | 108.418300 | 3.255448 |
| **T5707842** | 110.910300 | 10.148950 |
| **T5732024** | 131.256400 | 10.660150 |
| **T5732438** | 121.198300 | 13.470880 |
| **T5734136** | 124.638400 | 5.219774 |
| **T5734712** | 124.225700 | 11.508890 |
| **T5735247** | 92.000120 | 6.933465 |
| **T5752430** | 116.012900 | 3.801635 |
| **T5754768** | 112.942900 | 3.565794 |
| **T5763332** | 145.692600 | 9.400865 |
| **T5766912** | 142.676100 | 3.854452 |
| **T5767449** | 137.842500 | 8.289958 |
| **T5770103** | 134.451500 | 8.918651 |
| **T5772464** | 143.071800 | 11.434570 |
| **T5773180** | 136.297800 | 6.596486 |
| **T5788969** | 25.144740 | 2.268958 |
| **T5870629** | 104.004600 | 9.927626 |
| **T5886321** | 108.843200 | 6.165574 |
| **T5895250** | 55.294390 | 5.484151 |
| **T5903245** | 98.052460 | 9.757709 |
| **T5924388** | 102.416500 | 2.066115 |
| **T5966220** | 120.607500 | 2.339625 |
| **T5968594** | 117.988800 | 5.511988 |
| **T5969212** | 54.882990 | 0.678455 |
| **T5977279** | 130.061300 | 13.139390 |
| **T5977824** | 161.263700 | 8.289715 |
| **T5980002** | 130.383200 | 6.403899 |
| **T5980345** | 146.538300 | 1.138935 |
| **T5981328** | 134.815100 | 6.318796 |
| **T5983197** | 135.567100 | 2.592459 |
| **T5983619** | 143.297300 | 14.663490 |
| **T5994718** | 120.787200 | 21.776610 |
| **T5994994** | 133.164100 | 5.715116 |
| **T6001299** | 78.982120 | 9.651379 |
| **T6001913** | 82.579930 | 6.219836 |
| **T6001988** | 94.698470 | 3.678585 |
| **T6012822** | 116.572600 | 9.336194 |
| **T6016123** | 110.170400 | 5.677451 |
| **T6018482** | 78.537560 | 5.743603 |
| **T6026444** | 87.384780 | 4.670168 |

| | | |
|---|---|---|
| **T6029784** | 84.364130 | 12.535290 |
| **T6039878** | 98.732760 | 10.523790 |
| **T6050537** | 105.465100 | 6.022461 |
| **T6060880** | 90.847250 | 3.204049 |
| **T6068931** | 101.766700 | 3.519454 |
| **T6069152** | 120.421600 | 13.404610 |
| **T6079828** | 105.921700 | 8.691013 |
| **T6081064** | 86.134650 | 1.107347 |
| **T6102962** | 72.728670 | 8.179393 |
| **T6103630** | 101.261200 | 1.523837 |
| **T6104455** | 103.478600 | 11.013220 |
| **T6104892** | 123.407800 | 8.616337 |
| **T6167441** | 106.740100 | 6.514100 |
| **T6170169** | 106.088900 | 0.730741 |
| **T6174889** | 28.902810 | 3.305028 |
| **T6194892** | 108.663100 | 8.457677 |
| **T6204485** | 122.085100 | 11.918230 |
| **T6213688** | 103.710700 | 4.207651 |
| **T6218079** | 147.038200 | 11.659000 |
| **T6218960** | 105.385100 | 17.261890 |
| **T6224591** | 105.777000 | 4.267921 |
| **T6224758** | 105.607800 | 4.061787 |
| **T6225072** | 105.738300 | 6.543757 |
| **T6227178** | 129.318500 | 4.947039 |
| **T6227979** | 103.590300 | 3.639660 |
| **T6237671** | 105.515800 | 10.285700 |
| **T6242073** | 128.510600 | 2.726705 |
| **T6250371** | 104.823800 | 2.352713 |
| **T6254913** | 118.462000 | 6.227760 |
| **T6259554** | 112.102200 | 8.591616 |
| **T6287294** | 102.015700 | 2.818852 |
| **T6287930** | 106.319700 | 4.171897 |
| **T6304847** | 85.295680 | 9.700631 |
| **T6306281** | 98.546700 | 4.817472 |
| **T6309865** | 107.624800 | 5.255031 |
| **T6310179** | 112.907200 | 4.682452 |
| **T6312020** | 116.967900 | 2.371747 |
| **T6312701** | 118.632600 | 13.738910 |
| **T6323881** | 91.749600 | 2.899999 |
| **T6366504** | 103.196600 | 1.995736 |
| **T6405766** | 70.308660 | 7.099259 |
| **T6408891** | 59.418380 | 8.557070 |
| **T6411301** | 77.999010 | 7.554937 |
| **T6414714** | 77.964880 | 6.474469 |
| **T6416273** | 86.743410 | 8.930545 |
| **T6462346** | 96.148490 | 5.076025 |
| **T6466344** | 131.205500 | 1.647077 |
| **T6488647** | 43.571580 | 3.715671 |
| **T6497803** | 91.446870 | 3.836317 |
| **T6498887** | 92.590770 | 4.968485 |
| **T6503776** | 79.891320 | 3.685875 |
| **T6515486** | 106.121800 | 2.766665 |
| **T6518628** | 106.565500 | 3.910473 |
| **T6530228** | 90.623380 | 5.277973 |
| **T6542797** | 107.207000 | 6.686652 |
| **T6548861** | 109.117500 | 2.629512 |

| | | |
|---|---|---|
| **T6637040** | 104.155300 | 3.858261 |
| **T6644906** | 43.648640 | 7.203900 |
| **T6654242** | 80.246110 | 1.413876 |
| **T6655301** | 92.590790 | 12.786330 |
| **T6657824** | 42.002370 | 1.819481 |
| **T6697435** | 8.769226 | 4.212029 |
| **T6723459** | 88.549190 | 7.496999 |
| **T6735820** | 123.058500 | 4.328891 |
| **T6780107** | 18.238380 | 1.753272 |
| **T6781813** | 106.020000 | 4.821341 |
| **T6840609** | 87.715720 | 2.851618 |
| **T6842618** | 82.418820 | 6.142763 |
| **T6896665** | 92.035750 | 9.818319 |
| **T7007254** | 61.914710 | 9.312282 |
| **T7027113** | 75.231640 | 8.516233 |
